# Statistical Superiority Without Clinical Relevance: A Critical Appraisal of Recent Hyaluronic Acid Filler Studies

**DOI:** 10.1093/asj/sjaf139

**Published:** 2025-07-25

**Authors:** Jessica Hicks, Torun Bromée, Bill Andriopoulos

Over the past decade, the hyaluronic acid (HA) filler market has expanded significantly, making it increasingly important to critically evaluate the clinical data that substantiate claims of superiority of newer products over established filler technologies. A study by Kaminer et al was recently published, presenting data from a pivotal clinical study for 2 new HA fillers, EVL_F_ and EVL_S_ (Evolus Inc., Newport Beach, CA).^1^ Following a thorough review, we have concerns regarding the robustness of the statistical superiority claims, because they are based on a metric that lacks clinical relevance.

In this split-face nasolabial fold (NLF) clinical study, both EVL_F_ and EVL_S_ were compared with RES_L_ (Galderma, Uppsala, Sweden) for noninferiority. The authors claim statistical superiority of both products compared with RES_L_ based on their primary endpoint at 6 months: mean Wrinkle Severity Rating Scale (WSRS) score improvement assessed by a photographic reviewer panel. The differences in mean scores favoring their EVL products were −0.27 (Figure 1 in Kaminer et al, EVL_F_ vs RES_L_) and −0.22 (Figure 2 in Kaminer et al, EVL_S_ vs RES_L_), with statistical superiority supported by the confidence intervals not crossing zero and corresponding *P*-values.

Although the statistical data may appear to favor their products, we encourage readers to critically assess the clinical relevance of the small differences observed, which only amount to fractions of a point on a grading scale. Notably, the study was powered for a minimum of only 25 participants, yet nearly triple that number was enrolled in each arm (*n* = 70). This substantial increase in sample size prompts concerns about the potential for scientific misinterpretation when analyses are overpowered, because this may increase the likelihood of detecting statistically significant results even when the differences are minimal and lack meaningful clinical relevance.

First, we revisit the original publication of the WSRS score used in this study, in which the authors explicitly defined each grade as representing a clinically meaningful change in NLF severity relative to adjacent grades.^2^ Additionally, the FDA dermal filler panel recently discussed primary effectiveness responder rates, defined as the percentage of participants with at least a 1 grade improvement, as a benchmark for determining clinically significant outcomes.^3^ To align with these regulatory discussions, responder rates should be the standard for comparative claims, rather than mean changes from baseline expressed as fractional values, which lack meaningful clinical interpretation.

Given the absence of the change from baseline values for the “statistically superior” WSRS primary endpoint, our critique is reinforced by the underwhelming 6-month responder rates, with only about half (51.6%) or a minority (45.2%) of patients achieving a clinically meaningful improvement for EVL_S_ and EVL_F_, respectively. The authors also report statistically significant WSRS mean change scores favoring EVL_F_ over RES_L_ at all time points (Figure 3 in Kaminer et al), and EVL_S_ over RES_L_ at 6 and 9 months (Figure 4 in Kaminer et al), based on blinded evaluator ratings. Notably, the maximum difference between the groups was 0.4 points, a change that is unlikely to be perceptible or meaningful in a clinical context. Although the text notes that blinded evaluator responder rates were substantially higher than those from the photographic reviewer panel (primary endpoint), comparative data against RES_L_ were not published, focusing instead on individual ratings that lack clinical relevance. The decision to omit comparative responder rates raises our concerns about data transparency.

We also echo these concerns regarding 2 other recent publications of split-face NLF HA filler clinical studies.^4,5^ The first, a publication of the EVL_F_ European study (*n* = 45), reiterates claims of statistical superiority over RES_L_ at 3 and 6 months based on mean WSRS change scores, yet omits responder rate comparisons (Figure 1 in Lheritier et al).^4^ The second, a pivotal RHA4 study using LYFT as a comparator, reports statistical superiority of RHA4 (Teoxane SA, Geneva, Switzerland) vs LYFT (Galderma) at all time points (Weeks 24, 36, 52, and 64) based on blinded live evaluator (BLE)-assessed WSRS mean change scores, with a maximum difference of 0.18 points between both groups (Table 2, Figure 4A in Kaufman-Janette et al).^5^ Opposing these data, BLE-assessed WSRS responder rates (Figure 4C in Kaufman-Janette et al^5^) show statistical significance only at Week 24. The discrepancy between statistically significant mean change scores and responder rates reinforces our concerns about the increasing reliance on statistical outcomes that lack clinical relevance in evaluating HA filler efficacy.

In conclusion, although newer HA filler studies report statistical superiority based on mean change from baseline scores, the consistent omission of responder rates, an established benchmark for clinical relevance, raises concerns about the validity and transparency of these results. We urge the scientific community to rigorously scrutinize clinical data and base efficacy conclusions on outcomes that reflect meaningful clinical results, rather than placing undue emphasis on marginal statistical differences that lack practical relevance.

## References

[1] Kaminer MS, Avelar RL, Baumann L, et al Long-term safety and effectiveness of cold-crosslinked hyaluronic acid fillers: multicenter, randomized, controlled, double-blind study. Aesthet Surg J. 2025;45:842–849. doi: 10.1093/asj/sjaf080 40378267 PMC12260371

[2] Day DJ, Littler CM, Swift RW, Gottlieb S. The wrinkle severity rating scale: a validation study. Am J Clin Dermatol. 2004;5:49–52. doi: 10.2165/00128071-200405010-00007 14979743

[3] US Food and Drug Administration . FDA Executive Summary: General Issues Panel Meeting on Dermal Fillers. Published March 23, 2021. Accessed May 30, 2025. https://www.fda.gov/media/146870/download

[4] Lheritier C, Converset S, Rzany BJ, Cartier H, Ascher B. Efficacy of a new hyaluronic acid dermal filler on nasolabial folds correction: a prospective, comparative, double-blinded clinical trial. Dermatol Surg. 2024;50:746–751. doi: 10.1097/DSS.0000000000004207 38713883 PMC11288387

[5] Kaufman-Janette J, Taylor SC, Cox SE, et al Efficacy and safety of a new resilient hyaluronic acid dermal filler in the correction of moderate-to-severe nasolabial folds: a 64-week, prospective, multicenter, controlled, randomized, double-blind and within-subject study. J Cosmet Dermatol. 2019;18:1244–1253. doi: 10.1111/jocd.13100 31444861 PMC7384057

